# Implementing the ten steps to successful breastfeeding in multiple hospitals serving low-wealth patients in the US: innovative research design and baseline findings

**DOI:** 10.1186/1746-4358-8-5

**Published:** 2013-05-20

**Authors:** Miriam H Labbok, Emily C Taylor, Nathan C Nickel

**Affiliations:** 1 Carolina Global Breastfeeding Institute (CGBI), Department of Maternal and Child Health, Gillings School of Global Public Health, University of North Carolina, Chapel Hill, USA; 2 Manitoba Centre for Health Policy, Faculty of Medicine, University of Manitoba, Winnipeg, Manitoba, Canada

**Keywords:** Ten steps, BFHI, Breastfeeding, Multi-hospital, Operational research, Quality of care, Readiness to change

## Abstract

**Background:**

The Ten Steps to Successful Breastfeeding are maternity practices proven to support successful achievement of exclusive breastfeeding. They also are the basis for the WHO/UNICEF Baby-Friendly Hospital Initiative (BFHI). This study explores implementation of these steps in hospitals that serve predominantly low wealth populations.

**Methods:**

A quasi-experimental design with mixed methods for data collection and analysis was included within an intervention project. We compared the impact of a modified Ten Steps implementation approach to a control group. The intervention was carried out in hospitals where: 1) BFHI designation was not necessarily under consideration, and 2) the majority of the patient population was low wealth, i.e., eligible for Medicaid. Hospitals in the research aspect of this project were systematically assigned to one of two groups: Initial Intervention or Initial Control/Later Intervention. This paper includes analyses from the baseline data collection, which consisted of an eSurvey (i.e., Carolina B-KAP), Maternity Practices in Infant Nutrition and Care survey tool (mPINC), the BFHI Self-Appraisal, key informant interviews, breastfeeding data, and formatted feedback discussion.

**Results:**

Comparability was ensured by statistical and non-parametric tests of baseline characteristics of the two groups. Additional findings of interest included: 1) a universal lack of consistent breastfeeding records and statistics for regular monitoring/review, 2) widespread misinterpretation of associated terminology, 3) health care providers’ reported practices not necessarily reflective of their knowledge and attitudes, and 4) specific steps were found to be associated with hospital breastfeeding rates. A comprehensive set of facilitators and obstacles to initiation of the Ten Steps emerged, and hospital-specific practice change challenges were identified.

**Discussion:**

This is one of the first studies to examine introduction of the Ten Steps in multiple hospitals with a control group and in hospitals that were not necessarily interested in BFHI designation, where the population served is predominantly low wealth, and with the use of a mixed methods approach. Limitations including numbers of hospitals and inability to adhere to all elements of the design are discussed.

**Conclusions:**

For improvements in quality of care for breastfeeding dyads, innovative and site-specific intervention modification must be considered.

## Background

The US Surgeon General’s Call to Action to Support Breastfeeding [1] and the new Joint Commission for Hospital Assessment Perinatal Care Core Measure on exclusive breastfeeding [2] underscore the urgency to increase exclusive breastfeeding rates. Exclusive breastfeeding (EBF) is considered one of the most effective preventive health measures to reduce child morbidity and mortality, in the US and globally [3,4]. Nonetheless, there are considerable disparities in breastfeeding outcomes by socio-economic indicators and by race/ethnicity [1]. Low wealth populations in the US, as a group, demonstrate lower breastfeeding rates [5], and thus are vulnerable to higher incidence of breastfeeding preventable illnesses.

Ten practices called the ‘Ten Steps to Successful Breastfeeding’ were defined with suggested actions and evaluation question in 1989 [6]. When these practices are implemented by hospitals they have been shown to have a direct impact on breastfeeding initiation, duration and exclusivity rates [7]. The impact on rates of exclusive breastfeeding has been shown in assessments at the individual hospital level, at the national level and internationally [8-17]. The Baby-Friendly Hospital Initiative (BFHI) is a global program of designation of hospitals that have achieved full implementation of the Ten Steps as assessed using an internationally approved assessment tool [18].

The overall purpose of this project, which was developed prior to any hospital in North Carolina having achieved Baby-Friendly Hospital status, is to increase breastfeeding rates by helping women to achieve their breastfeeding intentions. While there are many assessments of implementation in individual hospitals, little has been done with multiple hospitals in low wealth settings to address the disparities mentioned above. Research has shown that supporting the quality of the hospital-based breastfeeding support services, and increasing the number of the Ten Steps practices in place will result in an increased number of women achieving their breastfeeding goals [19].

The research aspect of this project was undertaken to better understand how hospitals that serve low wealth populations and that have not necessarily considered seeking BFHI status might best be supported to implement all aspects of each of the Ten Step practices. This paper presents the study design and baseline findings from the research and from the non-research hospitals.

## Methods

### Project design

The Carolina Global Breastfeeding Institute (CGBI) Breastfeeding-Friendly Healthcare (BFHC) project identified low wealth hospitals, defined as with >60% clients’ income levels eligible for Medicaid. Efforts were made to include hospitals reflecting urban/rural, large/small hospitals, teaching/non-teaching hospitals, and those that were and were not planning to seek Baby-Friendly Hospital Initiative (BFHI) designation. A translational research approach was used within this project, with a quasi-experimental operational research design with mixed methods, as well as a cost analysis.

Translation of the achievements of basic science into everyday clinical practice remains a major issue in contemporary medicine, and is addressed through a new discipline, translational research, which aims to bridge the gap between basic and clinical research [20]. The term generally refers to the bench-to-bedside enterprise for harnessing knowledge from basic sciences to produce new treatment options, with the end point of production and use of a new technology [21]. The Canadian Institutes of Health Research (CIHR) define ‘knowledge translation’ in terms of exchange, synthesis, dialogue and interaction between researchers and users – a ‘radically different’ model from the unidirectional flow of knowledge sometimes implied by terms such as ‘dissemination’ or ‘knowledge transfer’ [22]. The key differences between the translational framework for public health research and the traditional linear translational medicine pathway include a redefinition of the endpoint (from the use of effective interventions to impact on population health), the incorporation of epidemiological approaches, inclusion of a wide range of biomedical, social and environmental sciences, and recognized interface with the public and their health decisions [23]. This may be akin to the difference between “going from ‘bench to bedside’ and “going from concept adoption to community adoption”.

Hospital selection and group assignment: staff reviewed available data from the 85 maternity centers [24] located across North Carolina’s six perinatal regions [25] to identify suitable hospitals. We invited participation by those hospitals that met the following criteria: 1) serve a low wealth population; 2) serve a racially/ethnically diverse population; and 3) report employing at least one International Board Certified Lactation Consultant (IBCLC). Hospitals meeting these criteria were contacted by telephone to assess the following additional selection criteria: 1) stated interest in improving quality of care in breastfeeding support, or in achieving the Ten Steps or seeking Baby-Friendly USA® designation; 2) expressed interest in participating, and 3) willingness to appoint a site coordinator to work with the project. Ten hospitals met these criteria, and of these ten, six agreed to be included in a comparative study of the impact of the intervention.

For inclusion in the overall project, we first sought to include at least one hospital in each of the six NC perinatal care regions to support understanding of implementation in various contexts; if there was only one hospital that fit the criteria in a region, it was included. Where there was more than one hospital in a region, an effort was made to include a mix of rural and urban hospitals and teaching and non-teaching hospitals across the state.

The final six study hospitals were then systematically assigned based on four hospital characteristics to create two similar groups. This assignment was based on 1) urbanicity, 2) size, 3) whether or not it was a teaching hospital, and 4) breastfeeding rate in the county where the hospital was located, creating the Phase 1 Group: Initial intervention, or Phase 2 Group: Initial control/delayed intervention. The remaining hospitals were invited to participate in project activities, share programmatic information and be informal opportunistic controls, but are not included in the research design. This paper includes an assessment of the effectiveness of this approach in achieving two comparable groups.

A phased quasi-experimental design was selected to allow comparison between intervention hospitals and non-intervention hospitals and to assess change over time [26,27]. Quasi-experimental design is similar to experimental design, however, the intervention and control groups are not created based on randomization of individuals, but rather on systematic or random allocation of groups [28]. Statistical analyses continue to use the individual as the basis of comparison. This design may be used in many health-care situations where naturally occurring groups exist. In this case, the entire group rather than the individual receives the experiment intervention, while the entire control group does not. Table 1 offers additional description of terminology related to this approach, as well as additional terms used throughout this article.

This model and timeline are presented, below. O indicates Observation, or data collection; X indicates Intervention; Subscripts indicate Phase. Phase 1 group received the intervention after the first round of data collection, while Phase 2 group received the intervention after the second round of data collection. The ‘Other’ hospitals were not part of the research; however, representatives were invited to several activities. The first 6 months included preparation, with identification of which hospitals would be included in Phase 1, with early intervention, Phase 2 to serve as control, with later intervention, or as Other, hospitals participating in the project, but not in the research cohorts. (Table 2)

### Consent

All participants in key informant interviews and/or surveys were administered approved informed consent, and results maintained in secure files during the project. The project and approach were approved by the UNC IRB.

Initial data collection, or ‘Observations’, O_1,_ include the results of administering three quantitative instruments, 1) the Carolina Breastfeeding Knowledge, Attitude, and Practice survey (Carolina B-KAP), (described below) 2) the Baby-Friendly USA® (BFUSA) Self-Appraisal Tool [29], and 3) the CDC Maternity Practices in Infant Nutrition and Care (mPINC) [30] survey to assess level of implementation of the Ten Steps. O_2_ and O_3_ are the follow-up data collection, using the same instruments and approach as in the baseline. The Self-Appraisal and mPINC instruments were selected as they are designed to elucidate not only current conditions, but also to help an institution identify what changes might be implemented to better achieve all of the Ten Steps. They both have been widely used, the Self-Appraisal tool is used within the Baby-Friendly USA activity, and the mPINC is used nationwide by the CDC. The Carolina B-KAP was designed to address additional questions to further address the objectives of the study.

Initial intervention, X_1,_ is designed to address gaps identified in the analysis of the baseline data. Group 1 intervention was an iterative process to ensure that the hospital-specific issues were addressed. Areas of emphasis included: 1) support for formation of a multi-level, multi-disciplinary taskforce, 2) planning inputs, based on findings from initial data collection, 3) encouragement to round and regularly review breastfeeding rates and progress, 4) selection among intervention options, including sensitization training for decision-makers, breastfeeding support self-efficacy and clinical skills training for nurses and physicians, support in outreach to community, and other step-specific support activities individualized to meet the specific needs of each hospital, and, 5) user-friendly materials developed to address barriers identified at baseline. Each area was reviewed with each contact, progress was evaluated, and adaptations instituted. During the first phase, the Group 2 - initial control/delayed intervention - and other hospitals received only feedback on the baseline data with recommendations for action. X_1_ is the continued support for phase one hospitals, while X_2_ is the intervention for Group 2, which was informed by the first phase, and developed to address any identified gaps and to reduce costs while maintaining efficacy. The other hospitals were also offered some support if requested based on the modifications and resources available.

In addition, all hospitals have subsequently been invited to participate in two statewide efforts in 2011: the Perinatal Quality Collaborative of North Carolina (PQCNC) [31] offers support for hospitals to address the new Joint Commission Perinatal core measure on exclusive human milk feeding during the maternity stay (CGBI/BFHC personnel are providing the technical inputs) and the NC Maternity Center Breastfeeding-Friendly Designation (NCMCBFD) [32]. NCMCBFD, offered by the State Division of Public Health and endorsed by the NC Hospital Association, is a designation process recognizing progress on the Ten Steps. CGBI/BFHC personnel are supporting these two efforts. Participation/non-participation in these efforts will be considered in analysis of data collected post initiation of these activities.

**Table 1:** Abbreviations and glossary

**BF(ing):**	**Breastfeed(ing)**
BFHC:	The CGBI Baby-friendly Healthcare activity
BFHI:	Baby-friendly Hospital Initiative, developed and supported by WHO and UNICEF
BFUSA®:	BFUSA® is the registered trademark of the US organization - Baby Friendly: United States of America - that carries out the designation process for maternity care settings, using a modification of the international BFHI guidelines
BFUSA Self-Appraisal	The BFUSA self-administered checklist that permits a facility to make an initial review of its policies and practices related to the Ten Steps. Completing this tool serves as a needs assessment for mapping out a work plan.
Carolina B-KAP:	The BFHC knowledge, attitudes and practices survey instrument
CGBI:	Carolina Global Breastfeeding Institute; Department of Maternal and Child Health; Gillings School of Global Public Health at the University of North Carolina, USA
EBF(ing):	Exclusive breastfeed(ing), or exclusively breastfed
eSurvey:	Electronic survey
IBCLC:	International Board Certified Lactation Consultant
KAP:	Knowledge, Attitudes and Practices
L&D:	Labor and Deliver
LDRP:	Labor, delivery, recovery and post-partum
Mother-baby:	LDRP care for both mother and baby by the same nurse
mPINC:	The Maternity Practices in Infant Nutrition and Care (mPINC) is a national survey of maternity care practices and policies that is conducted by the CDC every 2 years beginning in 2007. The survey is mailed to all facilities with registered maternity beds in the United States and Territories to be completed and returned to CDC
NC:	North Carolina
NCMCBFD:	North Carolina Maternity Center Breastfeeding-Friendly Designation recognizes North Carolina hospitals and birthing centers that adopt policies and practices from the Ten Steps to Successful Breastfeeding, supporting the initiation, continuation and exclusivity of breastfeeding, providing a star for every two steps in place. NCMCBFD is endorsed by the North Carolina Hospital Association and the North Carolina Child Fatality Task Force.
NICU:	Neonatal Intensive Care Unit
O:	Notation commonly used in operational research to indicate ‘observation’, or data gathering
ORC:	Organizational Readiness to Change
PedNSS:	Pediatric Nutrition Surveillance System
PICU:	Pediatric Intensive Care Unit
PQCNC:	Perinatal Quality Collaborative of North Carolina
Quasi-experimental research design	Similar to experimental research design, however, the unit of randomization may be groups rather than individuals. However, analytic approaches are the same as those used in experimental research. (See text)
Translational Research	Translational research “translates” basic science into treatment modalities. In public health research, the endpoint of the translation effort is at a population level. (See text)
X:	Notation commonly used in operational research to indicate ‘intervention’

The phased intervention study design also allows the project to consider which approaches were observed to have greater or lesser impact, and to amend the approach used in the second intervention phase. This will lend clarity to the issues specific to implementation in the selected hospitals, all of which serve low wealth populations.

**Table 2:** Project and study design and timeline

**Time for each activity**	**6 mo.**	**3mo.**	**12 mo.**	**3mo.**	**12 mo.**	**3mo.**	**9mo.**
Phase 1 Group	Preparation and	O_1_	X_1_	O_2_	X_1_	O_3_	Continued activities
Phase 2 Group	Group	O_1_		O_2_	X_2_	O_3_	Continued activities
‘Others’	Assignment	O_1_		O_2_		O_3_	Continued activities

A mixed methods research approach [33] was employed as we were focusing on research questions that call for real-life contextual understandings and multi-level perspectives and wished to both explore frequency of Ten Step knowledge, attitudes and practices, as well to explore both the meaning and the understanding of the Ten Steps in this context. Therefore, we also carried out a qualitative study including key informant interviews. This use of multiple methods (i.e., intervention trials, multiple measurement tools, and in-depth interviews) approach intentionally integrated and combined these methods to draw on the strengths of each in our interpretation, both for the planned mid-project modifications and to help examine what worked and what did not work within the intervention elements.

### Theoretical framework

The theory of reasoned action or planned behavior was the primary theory used to plan this study. A high correlation of attitudes and subjective norms to behavioral intention, and subsequently to behavior, has been confirmed in many studies. We posit that the interventions selected (e.g., addressing obstacles with ways to facilitate progress, information feedback, sharing, adult learning, multi-level taskforce support, and external support for breastfeeding knowledge, attitudes and practices (KAP), etc.) would suggest that supporting patients in breastfeeding is ‘positive’, leading to higher motivation to be supportive. This higher motivation would, in turn, lead to information-seeking and mother/baby-supportive behaviors, and take providers through pre-contemplation, contemplation, preparation, action and maintenance of the new care behaviors [21]. This is enhanced by the ‘behavioral interactivity’ considerations that lead to consideration, which suggest that change is dependent on interactions within the community of providers [34].

Individual Behavior Change (Stages of Change) [35] and Organizational Readiness to Change (ORC) theories also served as bases of the conceptual framework [36-38]. Organizational Readiness to Change (ORC) theory is a multi-level, multi-faceted construct related to a specific change effort, in order to advance both the practical and theoretical knowledge of hospitals’ processes. This theory includes the constructs of Collective Efficacy and Collective Commitment to implement the Steps; further described in related article [39].

### Definitions

Definitions of breastfeeding vary in the literature [40]. For simplicity in this paper, the term breastfeeding (BF) is used to describe both breastfeeding and human milk feeding as defined in documents of the Academy of Breastfeeding Medicine [41]. Exclusive breastfeeding (EBF) stipulates that no supplementation of any type (including infant formula, cow's milk, juice, sugar water, baby food and anything else, even water) except for vitamins, minerals, and medications is fed to the infant [42]. In this paper, hospital practices of breastfeeding and exclusive breastfeeding throughout the hospital stay i.e., from birth to discharge, generally at 36 to at about 48 hours postpartum, are derived from record review. In addition, an acronym key is provided as Table 1.

### Data collection instruments

#### Breastfeeding rates

As none of the hospitals in this study were found to have a searchable system of recording feeding patterns, chart reviews were completed by the site coordinator, under the direction of the project director at each facility. The sample included 300 consecutive births, or three months of records. This provides a reasonable sampling frame for comparison over time of approximately 1 out of 4 births, in the smaller hospitals, and 300 births in the larger hospitals. These were collected during about the same time period that the other observation data were gathered. In addition, data on breastfeeding rates at the county level from the CDC Pediatric and Pregnancy Nutrition Surveillance System (PedNSS) [43], were reviewed and considered as a proxy for local breastfeeding rates in the low wealth population, since they are based on clients attending the public health clinics in each county.

#### Carolina B-KAP

The CGBI breastfeeding eSurvey (Carolina B-KAP) includes knowledge, attitude and practice (KAP) questions selected/developed to reflect each of the Ten Steps ‘global criteria’, i.e., the expanded definition of each of the steps as found in the BFHI materials [18]. Most questions were derived from standardized instruments for assessing clinicians’ attitudes about breastfeeding, assessing knowledge on providing breastfeeding support, and measuring the provision of breastfeeding support [30,44,45]. The Carolina B-KAP was also designed to measure theoretical constructs of Collective Efficacy and Collective Commitment (Discussed in separate paper [27]). The Carolina B-KAP covered knowledge, attitudes and practices. Scores were calculated separately for clinical and non-clinical staff, such that all KAP questions were answered by clinicians; non-clinicians only answered knowledge and attitude questions. The instrument included skip patterns to ensure that providers were asked questions relevant to their field of practice. For example, if a provider indicated he/she was not involved in labor and delivery, then he/she was not asked questions about breastfeeding within the first hour after birth. Each respondent is weighted equally.

The survey includes 53 questions, including seven knowledge questions, which cover contraindications, basics of latch, supportive practices, indications for supplementation, and recommended duration of exclusive breastfeeding. The knowledge score is calculated as the number of questions correct. Attitude is measured using eight questions on a six-point Likert scale; questions cover the importance of breastfeeding for health outcomes, equivalence with formula, importance of rooming-in, perception of difficulties for mother, and whether hospital staff can influence breastfeeding. Each question was scored from one to six such that a higher score reflects greater breastfeeding support. The overall attitude score is calculated as the average of the eight attitude questions.

The practice questions addressed counseling and clinical actions separately. Counseling and other clinical practice questions are asked as “proportion of patients who receive” the indicated support, in 5% increments. The possible score of 20 indicates an answer of 95-100%, while a score of 1 indicates 0-5%. The counseling questions addressed counseling per se, counseling on milk expression, and teaching BF techniques and identification of feeding cues, while the clinical practice questions covered provision and use of supplementation, pacifiers, and nursery (vs. rooming in). The overall counseling and clinical practice scores are calculated as the average of the responses.

Factor analyses were conducted to assess the construct validity of the attitude, practice, and organizational readiness sections of the Carolina B-KAP. Analyses using Item Response Theory assessed the knowledge questions.

The overall score is calculated as the percent of total possible points for the four sections (knowledge, attitude, clinical practice, and counseling practice) combined. The score is presented as the percent breastfeeding-supportive based on the 53 possible points.

#### Key informant interviews

A semi-structured key informant interview guide was developed with primary questions, follow-up questions, and probes [46]. The guide included questions about barriers and facilitators and was designed to capture the staff perception and other aspects of the hospital’s readiness to pursue BFHI designation. The guide also explored readiness to implement each Step by including questions about specific practices that are the components of each of the Ten Steps. CGBI/BFHC staff reviewed the guide for face validity. The interview guide was then pilot-tested with two individuals who provide maternity care in non-project Hospitals. Based on the above, the CGBI/BFHC staff modified finalized the key informant interview.

Response to the BFUSA® Self -Appraisal Tool and the CDC Survey of Maternity Practices in Infant Nutrition and Care (mPINC) were collected, and contribute to the results presented here and in companion papers [37,46].

#### Self -appraisal tool (SAT)

The BFUSA^®^ Self-Appraisal Tool, used with permission, based heavily on the WHO/UNICEF Self-Appraisal Tool, provides an appraisal of each facility’s adherence to the Steps [18,48]. It is intended to be completed by a team of key management and clinical staff members. For the purposes of this study, the site coordinator and a breastfeeding interest group at each hospital completed the tool. It consists of a series of forty-seven yes/no questions about policies and practices specific to each Step.

#### CDC survey of maternity practices in infant nutrition and care (mPINC)

CGBI/BFHC used the CDC mPINC as a second measure of participating hospitals’ provision of breastfeeding support. The mPINC collects data on maternity center policies and practices that support breastfeeding. The mPINC is a hospital-level instrument completed by an individual selected by hospital administration as the person most familiar with infant feeding practices at each facility [30]. The mPINC instruments was completed either by the same team that completed the Self-Appraisal Tool at each hospital or by the individual most knowledgeable about the facility’s infant feeding practices. Only the 33 question stems and sub-questions designed to assess adherence to policies and practices reflecting the Ten Steps were included in analysis.

### Data collection

The hospital’s site coordinator invited and encouraged all maternity staff to complete the Carolina B-KAP using both on-line and hardcopy paper versions as necessary. Pizza lunch parties were offered to the two facilities collecting the greatest percentage of completed surveys as an incentive.

Key informant interviews were conducted with thirty-four respondents, selected by each site coordinator in collaboration with CGBI to explore practice and attitudes vis-à-vis the Steps. Purposeful sampling was used to ensure representation of those staff members responsible for implementation of breastfeeding-related practice change at each hospital [49]. Two research staff trained in qualitative research methods conducted interviews in a private room at each hospital, with one asking questions and the other taking notes, recording with a digital audio recorder, and asking follow-up questions when appropriate. Interviews lasted 30–50 minutes until achieving construct saturation. Table 3 presents selected questions used to explore practice and attitudes about the Ten Steps [38]. A professional transcriptionist created verbatim, typed records of the digitally recorded interviews. The second and third authors used a code book with decision rules to independently code and memo the transcripts in Atlas.TI [50]. They met and reviewed the coded transcripts to achieve consensual validation [51,52]. Findings were summarized and presented back to the hospitals for member-checking.

**Table 3:** **Summary of selected attitude and practice questions from the semi-structured key informant interview guide**[39]

**Primary questions**	**Secondary questions**
**• Could you please describe the current practice of these 10 Steps in your facility? ( *Walk the respondent through each of the Ten Steps. *)**	a. Does hospital policy reflect the Ten Steps? How is the policy communicated to staff? Communicated to patients? Is the policy posted?
	b. Who receives training for providing breastfeeding-supportive care?
	c. Does your facility have a prenatal class for patients? Is BF included in the prenatal class? Is there a specific breastfeeding class?
	d. How do staff support women to initiate BF w/in an hour? What does the staff do to help mom initiate? Are babies placed skin-to-skin? What does that look like?
	e. What do staff do to show women how to breastfeed? Who is mainly responsible for fulfilling this task? Do staff teach hand expression, how to pump?
	f. How often do breastfed infants receive something other than human milk? What about infants who stay primarily in the nursery?
	g. What happens at night re: rooming-in? How do moms respond to the idea of rooming-in?
	h. In general what do staff think “on-demand means”? What does on-demand mean to you? What are some of the cues that staff use to know when to feed the baby? What do staff teach mothers re: when to feed their baby.
	i. Are pacifiers readily available for babies? If a baby is not breastfeeding well what sorts of techniques do staff use to supplement the infant (ask about cup feeding, bottle feeding, other)?
	j. What does the facility do to foster the establishment of support groups? How does staff refer moms to support groups? What support is available in the community that you’re aware of?
**• Are there any barriers, here at your facility, that may make it more difficult to implement these 10 Steps?**	
**1. *(for each Step) *Could you explain for me your perceptions of Staff ability to work together to practice Step ____?**	a. What factors influence staff members’ ability to work
	together to implement this Step?
	b. What factors make staff members more able to practice the Step?
	c. What factors make staff members less able to practice the Step?
**2. Could you explain for me your perceptions of Staff commitment to work together to practice Step ____?**	a. What factors influence staff members’ commitment to work together to implement this Step?
	b. What factors make staff members more committed to practice the Step?
	c. What factors may lead staff members to be less committed to implement this Step?

The semi-structured key informant interviews were analyzed for themes and informed the intervention design. These interviews were repeated as one aspect of the “Observation” or data gathering at the end of the first and second phases of intervention.

Cost data, including implementation, training, and research costs, are maintained and verified with each facility and at CGBI. Detailed explanation of cost analysis will be provided in a companion paper.

### Analytic approach

Hospitals were systematically assigned and we used standard analysis approaches for quasi-experimental designs. Multiple-case study methods are used where cases (i.e., hospitals) are studied in-depth, longitudinally, using multiple data sources and types to explore the support approaches required for Step implementation [53].

Initial analyses explored whether there were any statistically significant differences between the two systematically assigned treatment groups using Chi-square contingency tables with two-sided Fisher’s exact tests. Significance was observed at p < 0.05. Analyses that used the hospital as the unit of study employed non-parametric Wilcoxon Ranked Sum test, and, given the small numbers of hospitals for the nonparametric tests, we set the *a priori* p-value cutoff at 0.10 for these analyses.

All quantitative analyses are being conducted in Stata/IC 10.1 software [54].

## Results

### Comparisons of the baseline characteristics of phase 1 and phase 2 hospitals

The six selected hospitals serve at least 60% low wealth clients, and vary in maternity center staff size, including nurses, physicians, administrators, and others, ranging from 50 to 400 employees. The number of annual births ranged from approximately 600 to 6,000 in 2008. Descriptive information on the factors used for systematic assignment for the groups of hospitals is indicated in Table 4, reflecting reasonable comparability given the number of hospitals included. The other group, which is the non-selected hospitals, is not included in the study design; however, baseline data are presented for discussion purposes.

Comparisons revealed no statistically significant difference between the Group 1 and Group 2 hospitals in the variables under study, with very similar findings for average number of births, percent of all statewide births, proportion urban, county level and hospital level breastfeeding rates, cesarean births, and completion of the study survey. Two variables, however, merit further discussion. First, the difference in proportion teaching/non-teaching hospitals was unavoidable to ensure at least one hospital in each region. Next, we asked that the hospital have at least one IBCLC; we found that there was a good deal of variation in the numbers, which was unexpected, however, all that were included in the study met the minimum that we requested.

Project-wide, 623, or slightly more than 50% of all eligible staff, returned a completed the Carolina B-KAP. Their characteristics reflect the effort made to include all categories of hospital staff whose work impacts maternity care. While respondents were 97% female, they represent a cross-section of staff impacting new mothers: 77% nurses, 7% physicians, 3% lactation services, 2% administrators, and 1% other, such as dieticians. In addition, they represented appropriate units: 59.4% Labor and Delivery (L&D), Labor, Delivery, Recovery and Postpartum (LDRP), or Mother-baby care units; 16% Neonatal/Pediatric Intensive Care Unit (NICU/PICU); 9% other wards, 2% administration, and 14% other. Racial/ethnic distribution was 87% White, 6% Black, 2% Latino, and 6% other.

The Carolina B-KAP covered knowledge, attitudes, and practices as described in the Methods section and results are presented as Table 4. Overall, Groups 1 and 2 respondents reflect similar levels of knowledge and attitudes at baseline with 94% of respondents stating that they can have a positive influence on mothers’ infant feeding practices. The scores for reported support practices reflect that an average of only about 60-65% of mother/baby dyads receive the support indicated. The overall scores were in the low 60s out of 100, demonstrating room for improvement. There was no statistical difference between the groups.

**Table 4:** Baseline characteristics of hospitals

	**Intervention**	**Control/ Delayed intervention**	**Comparison of intervention and control**	**Other hospitals (not included in research design)**
**Mean births per annum**	2684	2046	p = 0.51*	2316
**Proportion urban**	1/3	1/3	p = 0.80**	1/2
**Teaching hospitals**	2 of 3	1 of 3	p = 0.50**	2 of 6
**Average BF initiation rate in%, by primary country(ies) served**[43]	60	62	p = 0.08**	38
**Approx.% of NC births**	2	2	p = 0.50	2
**Mean maternity staff / Birth**	0.1	0.1	p = 0.12*	0.1
**Mean age of staff**	37	39	p = 0.49*	39
**% Maternity staff White**	84	73	p = 0.80*	82
**Cesarean birth rate**	29	31	p = 0.24*	28
**% BF at discharge**	65	58	p = 0.82*	12
**% EBF at discharge**	32	30	p = 0.83*	7
**% completing Carolina B-KAP**	50	53	p = 0.81**	52

Hospitals are separated into three groups: Intervention: Group 1 (Early intervention); Early control/later intervention: Group 2; and additional hospitals not included in research design: Other. The first 4 variables in this table were those used to systematically assign the hospitals to Group 1 or 2. The “Other” hospitals participated in selected meetings and trainings, but were not included in the study design.

*Two-sample Wilcoxon rank-sum (Mann–Whitney) test.

**Fisher’s exact test, 2-sided.

### Barriers and facilitators

All baseline data collection tools – qualitative and quantitative survey elements, key informant interviews and feedback discussions – were reviewed to identify factors seen as barriers and facilitators to making change and progress on the Ten Steps. These are presented in Table 5. While some barriers have been identified in the literature, e.g., older staff, the assumption that rooming-in would create patient dissatisfaction, other barriers were concerned with new issues, e.g., assumption that the Lactation Consultant (LC) would do all breastfeeding support, lack of self-efficacy among staff, expense of BFHI designation. The facilitating factors, or facilitators, were not yet in place in most settings. Examples include the difficulties in assessment of breastfeeding rates and related progress/statistics, opportunities for discussion, lack of management support, and lack of understanding of the benefits of breastfeeding and the skills to support it. The barriers and facilitators identified through all the baseline processes are presented in Table 5. The specific steps that were found to be associated with higher breastfeeding rates at baseline are reported in a companion article [48].

## Discussion

The Breastfeeding Friendly Healthcare (CGBI/BFHC) project was undertaken to support the implementation of the Ten Steps in low wealth settings to overcome hesitancy to implement the Ten Steps in North Carolina, and to explore the impact of a stepwise, interactive, and locally adapted approach, rather than an “all or nothing” standardized approach. Analysis of the baseline data reveals that the two intervention groups are comparable. However, a few unexpected differences were identified in the baseline data collection, including the mean number of IBCLCs and the distribution of teaching hospitals; while not statistically significant, these factors will be taken into account in future analyses.

One limitation identified early in the implementation of this study is that none of the hospitals were collecting and recording breastfeeding rates in a readily retrievable manner. Hence, none of the hospitals, or their clinical staff, was carrying out any review and discussion as to best action on these data and progress on a regular basis. These are issues that pertain well beyond the research hospitals, and the development of approaches to address these deficits will be considered in the interventions developed in the project. Breastfeeding rates were initially reported to be quite high, but after chart reviews were completed, the study facilities were not statistically significant or different from the rates reported among the low wealth clients who attend public health clinics in the area. This consistent internal misperception of the breastfeeding rates by facilities further confirms the need for better breastfeeding records.

The identified barriers and facilitators serve as the basis for intervention planning. In addition, the inter-hospital differences in infrastructure and management systems are being taken into account in individualization of approaches. As a result of the preliminary findings, Group 2 will introduce 1) modified materials, to include suggestions for improved data collection and suggested ways to place controls on commercial infant formula distribution, 2) facilitate communication between participating hospitals to enhance problem solving, especially where there are common issues, and 3) other modifications to reduce costs while maintaining impact.

Limitations of this study include 1) self-selection bias in that those hospitals interested in the subject matter are more likely to have responded and enrolled; 2) small numbers of hospitals in the research design due to resource constraints and level of interest at the time of proposal preparation; 3) high initial attitude scores in the instruments may limit the possibility of observing significant improvement in this parameter, and 4) the response rate of about 50%, while high for an eSurvey [55,56], may limit generalizability beyond the planned comparisons. Nonetheless, measurement of progress on the Ten Steps and in the rates of breastfeeding initiation and exclusivity in-hospital may offer more insight. Further, while the representativeness of these hospitals is limited by numbers and self-selection, the use of multiple case approaches in addition to primary quasi-experimental design will allow additional observations and reporting that may help inform those facilities attempting to increase exclusive breastfeeding during the hospital stay, whether for the Joint Commission measure or for overall quality of care, by implementing the Ten Steps.

**Table 5:** Perceived barriers and facilitators to progress on the ten steps

**Barriers**	**Facilitators**
• Older nurses and physicians	• Ready availability of in-hospital breastfeeding rates
• Staffing constraints: Need more LCs	• Rounding on progress/statistics
• Interference in mothers’ choices	• Opportunities for staff to discuss and consider
• Increasing C/S rate	• Advocacy for breastfeeding at multiple levels within the facility
• Assumptions re: Hispanic culture	• Strong management support for *Ten Steps*
• Lack of self-efficacy among nurses	• Creating an atmosphere of openness to changing practices
• Perception negative to rooming-in	• Emphasizing and demonstrating benefits of breastfeeding to nurses
• Perception physicians will oppose policy changes	Including breastfeeding support in personnel evaluations
• Expense of baby-friendly designation and budget constraints	• Seeing mothers utilizing lactation services
• Nights: Staff practices	• Hands-on training
• Perception that the LC alone is responsible	
• Too many visitors in L&D	
• Pacifiers are needed for “fussy” babies and for the transition periods	
• Rooming –in will create patient dissatisfaction	

The following barriers and facilitators emerge from the qualitative key informant interviews.

## Conclusions

As an in-depth multi-method, multi-hospital operational study to explore issues in the implementation of the Ten Steps to Successful Breastfeeding, this study may shed additional insight of use to implementation of these Steps, and of BFHI, in the United States. Findings confirm that the study design has included a comparable set of hospitals in Group 1 and Group 2. However, the baseline finding that there is a lack of regularly collected data on breastfeeding in all of the hospitals, both those included in the research and the others, is of major concern. Regular review of, or rounding on, breastfeeding data trends by health practitioners is a well-recognized intervention strategy in clinical settings; it can stimulate action for improvements in practices. This study also yielded an extensive listing of potential barriers and facilitators that vary among hospitals and that might be considered in future intervention efforts. These findings and approaches may be useful in initiation of discussion with other facilities striving to implement the Ten Steps to Successful Breastfeeding for better maternal and child health outcomes.

## Competing interests

The authors declare that they have no competing interests.

## Authors’ contributions

MHL: Served as PI, conceived, designed, and provided oversight for all elements of the project and study, and wrote sections prepared final draft. ECT: Served as Project Director, executed or oversaw all project activities, significant contribution to design of the project and technical inputs, planned qualitative data collection and handling, drafted parts of the paper. NCN: Carried out project activities, developed and tested the Carolina B-KAP, planned and carried out all statistical analyses, developed semi-structured interview guide, planned qualitative data collection methods, conducted qualitative data analyses, prepared tables, drafted the methods section. All authors participated in preparation of, and read and approved the final manuscript.
